# Expression of adipocytokines in heart fat depots depending on the degree of coronary artery atherosclerosis in patients with coronary artery disease

**DOI:** 10.1371/journal.pone.0248716

**Published:** 2021-03-18

**Authors:** Olga V. Gruzdeva, Ekaterina V. Belik, Yulia A. Dyleva, Daria A. Borodkina, Maxim Yu. Sinitsky, Danil Yu. Naumov, Evgeniya E. Bychkova, Elena V. Fanaskova, Elena I. Palicheva, Anastasia A. Kuzmina, Viktoriya N. Karetnikova, Olga L. Barbarash

**Affiliations:** Federal State Budgetary Institution “Research Institute for Complex Issues of Cardiovascular Disease”, Sosnovyi bulvar, Kemerovo, Russian Federation; The Ohio State University, UNITED STATES

## Abstract

In coronary artery disease (CAD) the adipocytokine content in the heart fat depot is altered, but it has not been established whether these changes are associated with the degree of atherosclerotic damage to the coronary artery (CA). Were examined 84 patients with CAD, and according to the degree of atherosclerotic state based on the SYNTAX Score scale, were divided: 39 moderate (≤22 points), 20 severe (23–31 points) and 25 extremely severe (≥32 points). Biopsies of subcutaneous (SAT), epicardial (EAT) and perivascular adipose tissue (PVAT) were obtained during elective coronary artery bypass grafting (CABG). The expression of adipocytokine was determined using real-time PCR. The concentration of the studied adipocytokines in adipocyte culture medium was measured by ELISA. Statistical analysis was performed using logistic regression analysis. In the adipocytes of the cardiac depot of patients with CAD, an increase in the expression and secretion of leptin and IL-6 and a decrease in adiponectin, with a maximum manifestation in severe and extremely severe CA lesions, was observed. EAT adipocytes were characterized by minimal expression of the adiponectin gene maximal gene expression leptin and IL-6 compared to SAT and PVAT adipocytes.

## 1. Introduction

Coronary artery disease (CAD) remains the leading cause of death in industrialized countries. Previous studies have shown a close relationship between CAD and obesity, but the mechanisms mediating this relationship are complex and not entirely understood [1]. Of particular interest is the visceral adipose tissue (VAT) located in the zone of the epicardium and coronary artery (CA), capable of exerting vaso- and paracrine effects on the vessels of the heart [2]. It is known that the immuno-metabolic status and the nature of the adipokine profiles of epicardial and perivascular adipocytes differ. It has been found that epicardial (EAT) adipocytes secrete higher levels of pro-inflammatory factors that affect energy metabolism, vascular function, immunological and inflammatory reactions. Perivascular adipocytes are characterized by the accumulation of less lipid droplets, and high biomolecules involved in the regulation of vascular tone and endovascular homeostasis [3].

Of particular interest is the relationship between CAD and adiponectin, the levels of which are significantly reduced in obese people. It is known that adiponectin has antidiabetic and antiatherogenic properties, and its low plasma level is associated with multivessel CA disease in men with CAD [4]. Leptin and interleukin-6 (IL-6) have proatherogenic effects, contributing to the development of classic risk factors for atherosclerosis including, arterial hypertension, diabetes mellitus (DM), endothelial dysfunction, inflammation and platelet activation [5]. It is assumed that leptin controls the secretion of chemokines and cytokines. In particular, it stimulates the secretion of interleukin 6 (IL-6), tumor necrosis factor (TNF)-α, and IL-1, which induces the synthesis and activates the aggregation *in vitro* of transforming growth factor (TGF)-β by endothelial cells, plasminogen activator inhibitor (PAI-1), and platelet P-selectin, which may also contribute to atherothrombosis [6].

It is not known whether there is an obvious associated between the progression of CAD and changes in the expression of adipocytokine genes in various heart fat depots. Notably, the results of this type of work can theoretically justify the development of new therapeutic targets.

### Objective

To identify the features of *ADIPOQ*, *LEP* and *IL-6* expression by EAT, perivascular adipose tissue (PVAT), and subcutaneous (SAT) adipocytes, depending on the degree of coronary lesions in CAD.

## 2. Materials and methods

### 2.1. General clinical characteristics of patients and distribution by groups

**Fig 1:**
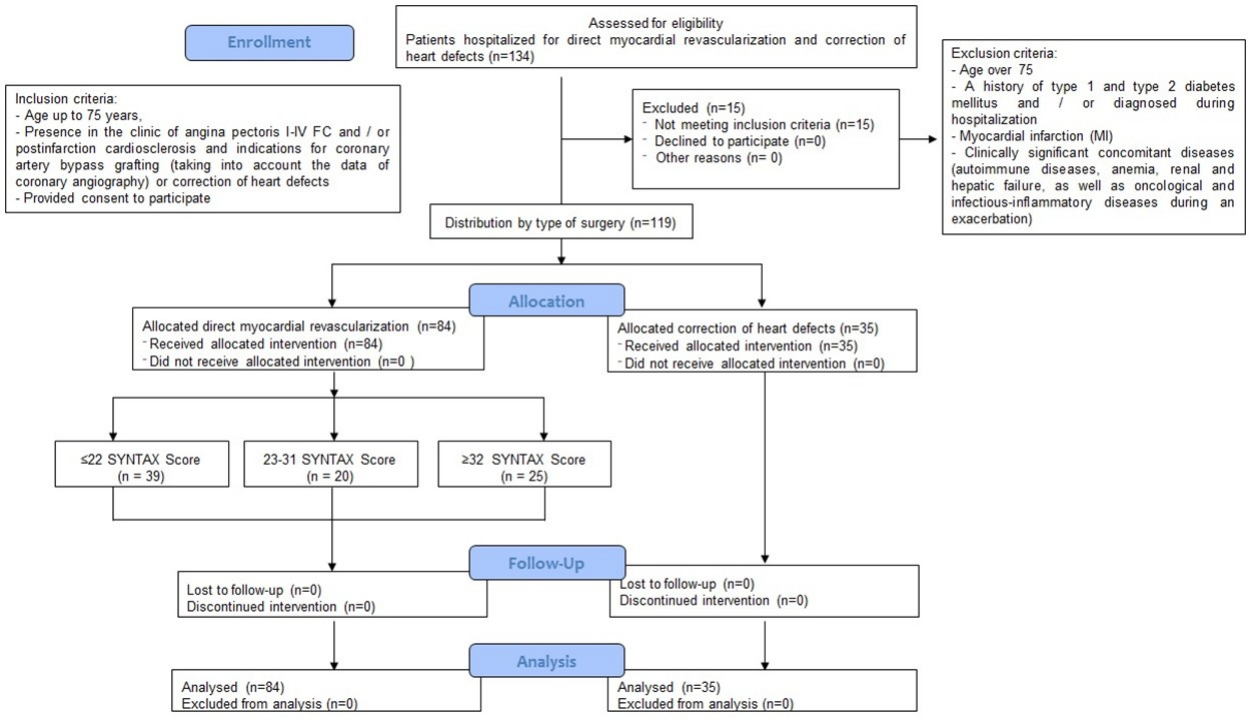
Flow chart with inclusion and exclusion criteria for patients in the study.

This retrospective study was carried out according to the Federal State Budgetary Institution Research Institute for Complex Issues of Cardiovascular Diseases. The research protocol was approved by the local ethics committee of the Research Institute for Complex Issues of Cardiovascular Diseases. Patients were recruited according to inclusion and exclusion criteria in 2017–2020 in accordance with the guidelines of good clinical practice and the principles of the World Medical Association’s Declaration of Helsinki (Ethical Principles for Medical Research with Human Participation), amended in 2000, as well as the "Rules of Clinical Practice in the Russian Federation" approved by the Order of the Ministry of Health of the Russian Federation (266/19.06.2003). All patients gave written voluntary informed consent prior to participation. A total of 84 CAD patients aged 64.6 (57.5; 68.5) years were examined. As a comparison group, 35 patients without CAD were included in the study according to the coronary angiographic study (CAG) (with acquired heart defects and indications for open surgery on heart valves; Fig 1). Inclusion criteria for persons with CAD were as follows: the presence of clinical angina pectoris functional class I-IV and/or post-infarction cardiosclerosis, the presence of indications for CABG, age up to 75 years, and consent provided for research.

The indications for CABG were:

- Presence of hemodynamically significant lesion of the main trunk of the left coronary artery (>50%) or the equivalent of a stem lesion, proximal lesion of all three vessels (>70%) or other lesions involving the proximal anterior descending artery.
- In patients with severe stable angina pectoris refractory to drug therapy, surgery was indicated for one- or two-vessel lesions without significant proximal stenosis of the anterior descending artery in the presence of a significant volume of viable myocardium
- Three-vessel lesion, complete functional revascularization at SYNTAX ≤22
- Three-vessel lesion, incomplete functional revascularization with SYNTAX >22
- Stenosis of the left trunk of the coronary artery (isolated or single-vessel lesion, orifice/trunk)
- Stenosis of the left trunk of the coronary artery (isolated or univascular lesion, bifurcation)
- Technically complex calcified lesion of the left trunk of the coronary artery
- Lesion of the orifice of the trunk of the left coronary artery and the circumflex artery, non-extended, multiple lesion
- Stenosis of the left trunk of the coronary artery and two- or three-vessel lesion, SYNTAX ≤32
- Stenosis of the left trunk of the coronary artery and two- or three-vessel lesion, SYNTAX ≤32
- High risk of bleeding due to concomitant pathology (hemorrhoidal, gastric ulcer), which excluded the use of two-component antiplatelet therapy during the time frame corresponding to clinical guidelines
- History of postinfarction cardiosclerosis with left ventricular aneurysm requiring resection

Inclusion criteria for persons with heart defects included the following: availability of indications for surgical correction of valvular pathology, age up to 75 years, and consent provided for research.

The study did not include patients that were >75 years with a history of type 1 and type 2 DM and/or myocardial infarction (MI) that was identified during examination during hospitalization, as well as a clinically significant concomitant pathology (autoimmune diseases, anemia, renal and liver failure, and oncological and infectious-inflammatory diseases during exacerbation). The examined individuals with CAD and heart defects received standard antianginal and antiplatelet therapy.

All patients underwent echocardiography (ACUSON Antares SIEMENS, Germany) as described by Judkins [7] on a two-projection Integris BH 3000 cardiovascular angiographic unit (Philips) using a Seldinger xenetic-350 puncture of the femoral or radial arteries. The presence of significant CA lesions was revealed and the lesions were classified as single-, or multi-vascular. This meant the presence of at least one significant stenosis in the projection of one to three main CAs (anterior descending, envelope and right coronary). The patients were, then, classified according to the degree of coronary lesion, determined by the SYNTAX scale using the online calculator (http://www.syntaxscore.com/): low (≤22, Group 1), intermediate (23–31, Group 2), and high (≥32, Group 3).

### 2.2. Sample collection and evaluation

We determined the expression of the *ADIPOQ* from biopsy samples (3–5 g of fat) obtained during surgery. The EAT samples were obtained from fat depots localized mainly around the right heart, while the SAT samples were from the lower angle of the mediastinal wound, and the PVAT samples were obtained from the mammacoronary vascular bundle in the anterior mediastinum, the ascending aorta. The resulting biopsies were placed in a Hanks balanced saline solution (SigmaAldrich, USA) with the addition of penicillin (100 units/l), streptomycin (100 mg/ml), gentamicin (50 μg/ml) and delivered to the laboratory. The adipocyte isolation from adipose tissue was carried out under sterile conditions in a Class II laminar cabinet (BOV-001-AMS MZMO; aseptic medical systems, manufacturer Miass Medical Equipment Factory, Russia) according to the procedure described previously [8].

### 2.3. RNA extraction

Total RNA was isolated from the adipocytes using the commercial RNeasy^®^ Plus Universal Mini Kit (Qiagen, Hilden, Germany) according to the manufacturer’s protocol with some modifications as described previously [9]. The quantity and quality of purified RNA were assessed using a NanoDrop 2000 Spectrophotometer (Thermo Fisher Scientific) by measuring the light absorbance at 280 nm, 260 nm, and 230 nm and calculating the 260/280 (A260/280) and 260/230 (A260/230) ratios. The integrity of the RNA was determined by electrophoresis in agarose gel, followed by visualization using the Gel Doc^™^ XR+ System (Bio-Rad, Hercules, CA, USA). Extracted RNA was stored at –70°C.

### 2.4. cDNA synthesis

Single-stranded cDNA was synthesized using the High-Capacity cDNA Reverse Transcription Kit (Applied Biosystems, Foster City, CA, USA) on a VeritiTM 96-Well Thermal Cycler (Applied Biosystems). Reverse transcription was performed using the program suggested by the kit’s manufacturer. The quantity and quality of synthesized cDNA were assessed using a NanoDrop 2000 Spectrophotometer. Samples were stored at –20°C.

### 2.5. Gene expression determination

Expression of the adiponectin (*ADIPOQ*), leptin (*LEP*) and *IL6* genes was evaluated by quantitative real-time polymerase chain reaction (qPCR) using TaqManTM Gene Expression Assays (ADIPOQ Hs00605917_m1, LEP Hs00174877_m1, IL6 Hs00174131_m1, Applied Biosystems, USA) on a ViiA 7 Real-Time PCR System (Applied Biosystems). Each 20-μL reaction mix contained 10 μL of TaqManTM Gene Expression Master Mix (Applied Biosystems), 1 μL of TaqManTM Gene Expression Assay (Applied Biosystems), and 9 μL of cDNA template (100 ng of cDNA + nuclease-free water) and was amplified under the following thermal cycling conditions: 2 min at 50°C, 10 min at 95°C, and 40 cycles of 15 sec at 95°C and 1 min at 60°C. As a negative control, we used 20 μL of reaction mix with no cDNA template. For each sample, a negative control, and three technical replicates were prepared. The results were normalized using reference genes *HPRT1*, *GAPDH*, and *B2M*. Test gene expression was calculated using the Pfaffl method and expressed on a logarithmic (log10) scale as a multiple change relative to the control samples.

### 2.6. Determination of cytokine and adipokine levels in the supernatant of cultured adipocytes

The concentrations of IL-6 and adipokines (adiponectin, leptin) in the culture medium was evaluated by enzyme-linked immunosorbent assay test systems (R&D Systems, Canada: D6050, DRP300, DLP00), according to the manufacturers’ instructions.

### 2.7. Quantification and statistical analysis

Statistical analysis was performed using GraphPad Prism 6 (La Jolla, CA, USA) and Statistica software, version 9.1 (www.statsoft.com, Tulsa, OK, USA). Nonparametric criteria were used to evaluate and analyze the data obtained. Comparison of two independent groups was carried out using the nonparametric Mann-Whitney test. Differences between the three groups were compared using one-way analysis of variance (ANOVA) for continuous variables. Categorical variables are expressed as percentages and were compared using chi-squared test or Fisher’s exact test. A P-value of <0.05 was considered statistically significant. A critical level of significance in testing statistical hypotheses was taken to be <0.05. Predictors of CAD progression were determined using one-dimensional polynomial logistic regression analysis and Area Under the Curve (AUC) definitions. The group with the absence of damage (Group 0) was used as a reference in the subsequent multivariate analysis for the phased inclusion of variables that were significant following the one-dimensional analysis. The results are presented as median, upper and lower quartiles (Me, Q1; Q3).

## 3. Results

Clinical and anamnestic characteristics of patients are presented in Table 1. Among the CAD cases, predominantly men had a history of arterial hypertension, smoking, and angina. Patients with heart defects had a minimal percentage of cardiovascular risk factors (Table 1).

**Table 1:** Clinical and anamnestic characteristics of patients.

Parameter	Patients with CAD, n = 84	Patients without CAD, n = 35	P
Men, n (%)	63 (75.0)	25 (71,4)	
Age, median (range), years	64.6 (57.5;68.5)	60.5 (54.0;65.1)	
BMI, median (range), kg/m^2^	29.43 (26.32;31.61)	26,59 (23.44;28.31)	
Excess body weight, n (%)	28 (33.3)	10 (28.6)	
Arterial hypertension, n (%)	81 (96.4)	8 (22.9)	P = 0.003
Hypercholesterolemia, n (%)	20 (23.8)	4 (11.4)	P = 0.024
Smoking, n (%)	58 (69.0)	10 (28.6)	P = 0.0001
***Anamnesis***			
Family history of CAD, n (%)	50 (59.52)	11 (31.4)	P = 0.018
Clinic of angina before the development of MI, n (%)	74 (88.09)	0	-
History of MI, n (%)	57 (67.85)	0	-
History of stroke, n (%)	6 (7.14)	0	-
CHF, n (%)	16 (19.0)	28 (80.0)	
CHF I FC, n (%)	8 (9.5)	4 (11.4)	P = 0.067
CHF II FC, n (%)	5 (6.0)	14 (40.0)	P = 0.002
CHF III FC, n (%)	3 (3.5)	10 (28.6)	P = 0.007
CHF VI FC, n (%)	0	0	-
Atherosclerosis of the 1^st^ CA, n (%)	6 (7.14)	0	-
Atherosclerosis of the 2^nd^ CA, n (%)	4 (4.76)	0	-
Atherosclerosis of three or more CA, n (%)	74 (88.1)	0	-
Atherosclerosis of other pools, n (%)	13 (15.48)	0	-
Ejection fraction, median (range), %	50 (43.0;56.0)	52.5 (43.3;57.5)	
Creatinine, median (range), mmol/L	93.2 (62.4;145.5)	97.1 (65.3;147.8)	

**Note:** P—level of statistical significance. **Abbreviations**: BMI–body mass index; CAD–coronary artery disease; MI–myocardial infarction; CHF–chronic heart failure; CA–coronary artery; FC–functional class.

The clinical characteristics of the examined individuals with varying degrees of coronary lesions are presented in Table 2. Overweight patients were found mainly in Groups 1 and 3 (Table 2). The percentage of smokers prevailed in Group 2. Group 2 patients often recorded cases of stroke and heart failure in their anamnesis, and one, two vascular lesions of the CA compared with other groups. Patients in Groups 1 and 3 did not differ in the frequency of occurrence of multivascular lesions of the CA among themselves, while persons in Groups 2 and 3 had statistically significant differences. According to other characteristics, the studied groups did not statistically significantly differ (p≥0.05).

**Table 2:** Clinical and medical history of patients with coronary artery disease depending on the degree of coronary lesion.

Parameter	Group 1 n = 39	Group 2 n = 20	Group 3 n = 25	P
Men, n (%)	27 (69.23)	17 (85.0)	19 (76.0)	
Age, median (range), years	64.1 (60.0;66.5)	65.2 (43.0;56.2)	65.1 (61.0;68.2)	
BMI, median (range), kg/m^2^	29.42 (29.15;30.22)	26.33 (25.21;27.44)	29.41 (29.29;31.68)	
Excess body weight, n (%)	15 (38.5)	3 (15.0)	10 (40.0)	P_1,2_ = 0.001
				P_1,3_ = 0.011
				P _2,3_ = 0.003
Arterial hypertension, n (%)	36 (92.30)	20 (100)	25 (100)	
Hypercholesterolemia, n (%)	10 (25.62)	4 (20)	6 (24)	
Smoking, n (%)	26 (66.67)	19 (95)	13 (52)	P _1,2_ = 0.011
				P _2,3_ = 0.013
***Anamnesis***				
Family history of CAD, n (%)	19 (48.75)	14 (70)	16 (64)	P _1,2_ = 0.023
				P _1,3_ = 0.014
Clinic of angina before the development of MI, n (%)	36 (92.31)	17 (85)	21 (84)	
Family history of CAD, n (%)	21 (53.85)	14 (70)	22 (88)	P _1,2_ = 0.023
				P _1,3_ = 0.024
History of stroke, n (%)	0	6 (30)	0	
CHF, n (%)	30 (76.92)	20 (100)	16 (64)	P _1,2_ = 0.023
				P _2,3_ = 0.021
Atherosclerosis of the 1^st^ CA, n (%)	3 (7.74)	3 (15)	0	
Atherosclerosis of the 2^nd^ CA, n (%)	0	4 (20)	0	P _1,2_ = 0.015
Atherosclerosis of three or more CA, n (%)	36 (92.33)	13 (65)	25 (100)	
Atherosclerosis of other pools, n (%)	6 (15.42)	3 (15)	4 (16)	P _2,3_ = 0.021
Ejection fraction, median (range), %	55 (45.0;57.0)	52 (48.0;55.0)	46 (38.0;56.0)	
Creatinine, median (range), mmol/L	83.5 (62.4;99.1)	94.74 (73.3;119.7)	101.47 (87.5;145.5)	P _1,3_ = 0.001

**Note:** P—level of statistical significance; P 1,2 –Groups 1 and 2; P 1,3 –Groups 1 and 3. **Abbreviations**: CA–coronary artery; CAD–coronary artery disease; CHF–chronic heart failure; BMI–body mass index; MI–myocardial infarction.

Our results show that in patients with CAD, the gene expression and adiponectin concentration decreased, while leptin and IL-6 increased in all fat depots with maximum deviations in cardiac localization adipocytes.

In CAD patients, *ADIPOQ* expression in adipocytes of heart fat depots was significantly lower compared to the same indicator in individuals without CAD: 2.10 (1.55;2.68) vs. 2.41 (2.19;3.09) in the EAT, and 3.11 (1.77;4.39) vs. 3.62 (3.23;4.62) in the PVAT. In contrast, the level of *LEP* increased in CAD patients and was higher in the EAT (1.40 [0.87;1.70] vs. 0.75 [0.57;1.19]) and the PVAT (0.91 [0.58;1.19] vs. 0.46 [0.34;0.68]). Similar results were observed for the expression of *IL-6* in the EAT and PVAT of CAD patients which increased to 0.080 (0.062;0.084) vs. 0.058 (0.037;0.073) and 0.058 (0.038;0.066) vs. 0.030 (0.021;0.032), respectively.

The impaired gene expression was accompanied by a change in the concentration of adipocytokines in the adipocyte culture medium *in vitro*. The adiponectin concentration in the adipocyte culture medium in the EAT of CAD patients was lower than in individuals without CAD (12.36 [11.65;14.47] ng/ml vs. 16.95 [14.34;18.56] ng/ml), and the same trend was observed for the PVAT medium (11.25 [10.72;13.96] ng/ml vs. 20.4 [17.84;23.04] ng/ml). The concentration of leptin in the culture medium of adipocytes was 7.67 (5.95;7.99) ng/ml and 6.96 (6.54;7.12) ng/ml for EAT and PVAT, respectively, in patients with CAD. This exceeded the level of individuals without CAD (EAT: 6.21 [4.33;7.34] ng/ml and PVAT: 4.50 [2.81;5.78] ng/ml). The concentration of IL-6 in the culture medium of adipocytes of cardiac localization of patients with CAD was higher than in individuals without CAD: 30.77 (20.11; 27.22) pg/ml vs. 22.26 (17.45; 25.12) pg/ml for EAT and 16.92 (13.38; 20.16) pg/ml vs. 12.49 (9.71; 17.17) pg/ml for PVAT. In the SAT of patients with CAD the expression and concentration of adipocytokines tended to decrease for adiponectin and increase for leptin relative to the comparison group (p>0.05).

The expression of adipocytokine genes and their concentrations in adipocytes of the adipose tissue localized in the heart depended on the degree of atherosclerotic lesion of the coronary lesion (Table 3).

**Table 3:** Expression of *ADIPOQ*, *LEP* and *IL-6* in epicardial and perivascular adipocytes that depended on the degree of coronary lesion.

Parameter	Adipocyte localization		Group 1 n = 39	Group 2 n = 20	Group 3 n = 25	P
			1	2	3	
Adiponectin gene expression, Delta Ct	EAТ	а	2.09 (1.91;3.87)	1.43 (1.15;2.58)	1.11 (1.03;2.42)	P_1.2_ = 0.013
						P_1.3_ = 0.005
	PVAТ	b	3.58 (3.01;4.63)	3.11 (1.93;4.11)	2.75 (2.27;3.62)	P_1а.1b_ = 0.0003
						P_2a.2b_ = 0.0003
						P_3a.3b_ = 0.0022
						P_1.2_ = 0.002
						P_1.3_ = 0.008
Leptin gene expression, Delta Ct	EAТ	а	0.68 (0.35;1.00)	1.47 (0.77;1.74)	0.76 (0.48;1.12)	P_1.2_ = 0.012
						P_2.3_ = 0.001
	PVAТ	b	0.66 (0.26;0.98)	0.75 (0.35;1.15)	0.90 (0.67;1.37)	P_2a.2b_ = 0.003
						P_1.3_ = 0.003
IL-6 gene expression, Delta Ct	EAТ	а	0.032 (0.026;0.045)	0.076 (0.043;0.096)	0.081 (0.054;0.097)	P_1.2_ = 0.003
						P_1.3_ = 0.012
	PVAТ	b	0.028 (0.014;0.033)	0.060 (0.037;0.086)	0.053 (0.034;0.086)	P_1.2_ = 0.003
						P_1.3_ = 0.004

**Note:** P—level of statistical significance; P 1,2 –Groups 1 and 2; P 1,3 –Groups 1 and 3. **Abbreviations:** EAT–epicardial adipose tissue; IL-6 –interleukin-6; PVAT–perivascular adipose tissue.

The *ADIPOQ* expression was maximal in Group 1 in all studied fat depot types compared to Groups 2 and 3. With an increase in the degree of CA lesion, *ADIPOQ* expression decreased. It should be noted that in the EAT the *ADIPOQ* expression was lower than in the PVAT at all stages of atherosclerotic lesion of the coronary channel (1.7 times in Group 1, 2.1 times in Group 2, and 2.5 times in Group 3).

In SAT the *ADIPOQ* expression was 2.73 (2.37;4.57) in Group 1, 2.25 (2.08;2.67) in Group 2, and 2.20 (2.10;2.58) in Group 3, which was higher than in the EAT, but lower than in the PVAT.

With an increase in the degree of lesion of the coronary lesion, gene expression and adiponectin concentration in the SAT decreased. In Group 1 the adiponectin mRNA level and its concentration in the SAT exceeded the same indicator in the EAT by 1.3 times in Groups 2 and 3 by 1.5 and 2 times, and its concentration—by 1.2 and 1.3 times, respectively.

In contrast to *ADIPOQ*, *LEP* expression was lower in Group 1. With an increase in the degree of atherosclerosis, *LEP* expression increased. In the EAT, a maximum expression of *LEP* was detected in Group 2: an increase of 2.2 times compared with Group 1, and 1.9 times compared with Group 3. In the PVAT, a high expression of *LEP* was observed in Group 3 individuals: an increase of 1.4 times relative to Group 1. Moreover, in Group 2 in adipocytes of the EAT, *LEP* expression exceeded 2 times its expression in the PVAT.

The expression of *IL-6* in fat depots of the heart increased with an increase in the degree of coronary lesion. Thus, the concentration of *IL-6* mRNA in adipocytes of the EAT in Group 2 and 3 patients exceeded that of individuals in Group 1 by 2.4 and 2.5 times, respectively, and that in adipocytes of the PVAT, by 2.1 and 1.9 times. In addition, the expression of *IL-6* in adipocytes of the EAT was 1.5 times higher than in the PVAT in Group 3.

Along with expression, the concentration of adipocytokines also depended on the degree of damage to the coronary lesion (Table 4).

**Table 4:** The concentration of adiponectin, leptin and IL-6 in the culture medium of epicardial and perivascular adipocytes, depending on the degree of coronary lesion.

Parameter	Adipocyte localization		Group 1 n = 39	Group 2 n = 20	Group 3 n = 25	P
			1	2	3	
Adiponectin, ng/ml	EAТ	а	12.36 (10.21;13.84)	10.47 (7.89;12.18)	8.10 (5.62;10.33)	P_1.2_ = 0.022
						P_1.3_ = 0.001
	PVAТ	b	11.25 (9.21;14.55)	9.38 (8.14;12.00)	9.23 (857;11.83)	P_1a.1b_ = 0.002
						P_2a.2b_ = 0.0014
						P_3a.3b_ = 0.003
						P_1.2_ = 0.0011
						P_1.3_ = 0.007
Leptin, ng/ml	EAТ	а	5.61 (3.95;7.00)	8.67 (6.35;9.92)	5.77 (3.98;7.17)	P_1.2_ = 0.032
						P_2.3_ = 0.0013
	PVAТ	b	3.96 (3.26;4.98)	7.95 (3.69;8.81)	6.96 (5.26;7.98)	P_2a.2b_ = 0.001
						P_1.3_ = 0.0032
IL-6, pg/ml	EAТ	а	15.92 (13.22;20.16)	28.17 (25.31;33.69)	30.77 (20.10;37.97)	P_1.2_ = 0.0041
						P_1.3_ = 0.022
	PVAТ	b	8.33 (5.45;10.01)	16.92 (13.37;20.19)	15.53 (13.05;19.64)	P_1.2_ = 0.0023
						P_1.3_ = 0.0012

**Note:** P—level of statistical significance; P 1,2 –Groups 1 and 2; P 1,3 –Groups 1 and 3. **Abbreviations:** EAT–epicardial adipose tissue; IL-6 –interleukin-6; PVAT–perivascular adipose tissue.

The highest adiponectin concentration in the culture medium of adipocytes of cardiac fat depots was in Group 1 patients compared to Groups 2 and 3: EAT (1.2 and 1.5 times, respectively) and PVAT (1.2 and 1.2 times, respectively). As atherosclerosis progressed, the concentration of adiponectin in the pericardial VAT decreased and was minimal in Group 3.

Comparative analysis of the groups showed that Group 2 was characterized by high gene expression, and an elevated level of leptin in the adipocyte culture both in the EAT and PVAT (1.6 and 2 times, respectively, in comparison with Group 1; 1.5 and 1.1 times, respectively, in comparison with Group 3). The concentration of leptin in the culture medium of the EAT exceeded that of the PVAT in Groups 1 and 3 by 1.4 and 1.1 times, respectively.

In the SAT, the expression and concentration of leptin was lower than in adipocytes localized in fatty deposits of the heart. Thus, the expression of *LEP* in the SAT was 0.30 (0.20;1.16) in Group 1, 0.77 (0.44; 1.77) in Group 2, and 0.33 (0.22; 1.14) in Group 3, which was significantly lower than in the EAT. The expression of *LEP* in the PVAT was higher than that in the SAT in Groups 1 and 3 (2.2 and 2.73 times, respectively).

The concentration of leptin in the SAT was 4.31 (3.92; 6.16) ng/ml, 7.57 (6.24; 8.38) ng/ml, and 4.33 (3.94;6.21) ng/ml in Groups 1, 2 and 3, respectively. The concentration of leptin in the PVAT in Group 1 was reduced relative to the SAT and increased in Groups 2 and 3 by 1.1 and 1.6 times, respectively.

The concentration of IL-6 in the culture medium of adipocytes of the EAT and PVAT in Group 1 decreased by 1.8 and 2 times, respectively, compared with Group 2 and 1.9 times, respectively, compared with Group 3. The concentration of IL-6 in the culture medium of adipocytes of the EAT exceeded that of the PVAT (by 1.9 times in Group 1, 1.7 times in Group 2, and 2 times in Group 3).

It should be noted that the increase in gene expression and IL-6 concentration was maximal in adipocytes localized around the heart, as compared with the SAT. Expression of *IL-6* in heart fat depots compared to the SAT increased by 2–2.5 times in the EAT and by 1.6–1.7 times in the PVAT in Groups 2 and 3.

Following logistic regression analysis, the predictors of multivascular lesions in CAD are a decrease in expression of *ADIPOQ* in the EAT (odds ratio [OR] 0.753, 95% confidence interval [CI], 0.650–0.985; area under the curve [AUC], 0.86; p <0.001) and the PVAT (OR, 0.893; 95% CI, 0.723–0.992; AUC, 0.88; p <0.05), a decrease in the left ventricular ejection fraction (LV EF) (OR, 0.955; 95% CI, 0.811–0.979, AUC 0.83; P<0.011), and an increase in *IL-6* expression in the EAT (OR, 2.846; 95% CI, 1.512–5.367; AUC, 0.89; p <0.001) and the PVAT (OR, 1.654; 95% CI, 1.113–3.271; AUC, 0.91; p <0.001). Additionally, the risk of developing atherosclerosis was lower in women compared with men (OR, 0.197; 95% CI, 0.170–0.563; AUC, 0.87; p <0.013) (Table 5).

**Table 5:** Logistic analysis results.

Parameters	OR	95% CI	P	AUC
*ADIPOQ* EAT	0.753	0.650–0.985	P<0.001	0.86
*ADIPOQ* PVAT	0.893	0.723–0.992	P <0.05	0.88
*IL-6* EAT	2.846	1.512–5.367	P<0,001	0.89
*IL-6* PVAT	1.654	1.113–3.271	P<0.001	0.91
LV EF, %	0.955	0.811–0.979	P<0.011	0.83
Women	0.197	0.170–0.563	P<0.013	0.87

Thus, in CAD, the gene expression and adiponectin concentration decreased, and the expression and concentration leptin and IL-6 in fat depots increased, with maximum deviations in the pericardial adipocytes. In Group 1 a higher level of adiponectin and a low content of leptin and IL-6 was observed. With an increase in the degree of atherosclerotic lesion of the coronary bed, the expression and concentration of adiponectin decreased and the expression and levels of leptin and IL-6 increased. The maximum changes in leptin were observed in the EAT (Group 2) and PVAT (Group 3).

## 4. Discussion

Our results showed that in CAD patients, the expression and concentration of adiponectin in adipocytes of the EAT and PVAT decreased compared to patients without CAD. A deficiency of adiponectin in adipocytes of cardiac localization is a pathognomonic sign of CAD due to coronary atherosclerosis. This has been confirmed by the results of experimental studies, which highlighted the ability of adiponectin to limit the process of atherogenesis. Adiponectin is known to inhibit macrophage uptake of oxidized low-density lipoproteins, causes the production of metalloproteinases, reduces the destruction of the fibrous coat of atherosclerotic plaque and neoangiogenesis, reduces the expression of adhesion molecules, and activates TNF-α (induced TNF-α-activation inhibits apoptosis of endotheliocytes) [10].

We have shown that as atherosclerosis progresses, the expression and thus the concentration of adiponectin decreases significantly and becomes minimal with extremely severe atherosclerosis. The results are consistent with data on the role of adiponectin in the progression of atherosclerotic lesions of the CA [11].

There is evidence of an inverse correlation of adiponectin levels with the progression of CA calcification. In addition, a mutation of the adiponectin gene was associated with its deficiency and with the development and progression of CAD [12]. Logistic regression results also confirm our assumptions. Low adiponectin mRNA levels in heart fat depots have been found to predict an increased risk of multivascular damage. Moreover, in combination with IL-6, the prognostic significance of adiponectin, regarding the risk of developing multivascular lesions in CAD, increased. We hypothesize that this may be due to suppression of the expression of *ADIPOQ* and the synthesis of adiponectin under the influence of increased pro-inflammatory IL-6 in heart fat depots. It is known that IL-6 is able to suppress the expression of *ADIPOQ*, not only in the SAT adipocytes, but also in the EAT and PVAT, without affecting the oligomerization of adiponectin. This hypothesis is supported by the fact that, in patients with CAD, the expression of the *IL-6* gene in the EAT is higher than in the SAT [13].

Indeed, in our study, the expression of the *IL-6* gene in fat depots of the heart increased with an increase in the degree of lesion of the CА. In addition, an increase in the level of IL-6 mRNA in the EАT and PVАT was a predictor of multivascular damage in CAD. Compared with the SAT, the expression of the *IL-6* gene in extremely severe atherosclerosis was increased by 1.7 times in the PVAT and by 2.5 times in the EAT. These findings are consistent with those of Chen K.H. et al. regarding the high concentrations of IL-6 in the EAT of patients with CAD compared with people without significant coronary stenosis, according to preoperative CAG, who underwent open heart surgery, including valve replacement, correction of ventricular, and atrial septal defects [14].

Previous studies have suggested that an increase in the level of IL-6 in the blood is more adequate than an increase in the concentration of C-reactive protein, and that it reflects the risk of multiple atherosclerotic lesions of the CA [15]. Our data indicate that the expression of the *IL-6* gene in adipocytes is important in the implementation of its pro-inflammatory properties at the systemic level. The expression of *IL-6* is characteristic of mature adipocytes, in contrast to their undifferentiated progenitor cells. The secretion of IL-6 in adipocytes is activated by β-adrenergic activation and is moderately reduced by glucocorticoids, while IL-6 itself stimulates lipolysis. The concentration of IL-6 in the АT is hundreds of times higher than in plasma, which suggests its important auto- and paracrine regulatory functions [16].

The increase in *IL-6* expression in adipocytes and its relationship with the degree of atherosclerotic lesion of the CA can be interpreted as a negative factor. IL-6 expression was found in the areas of the vascular bed most susceptible to atherosclerotic damage including the CA, cerebral vessels, and peripheral arteries.

The negative effect of IL-6 is manifested by a hypertrophic effect on cardiomyocytes. Hyperproduction of IL-6 is associated with angiotensin II, which, in turn, is able to stimulate the synthesis of IL-6 and is actively involved in the regulation of vascular inflammation, and the development and progression of atherosclerotic vascular damage. In addition, the protective effect of IL-6 on cardiomyocytes, which is manifested by an antiapototic effect, is also known [17]. IL-6 performs many positive functions by regulating the energy balance of the АT, and the intake of free fatty acids (FFA). FFA in the АT, and activating immune mechanisms, as well as limiting the inflammatory response by inhibiting the synthesis of pro-inflammatory cytokines, including TNF-α [18].

According to the literature, IL-6 secretion is stimulated by leptin [6]. The interconnection between IL-6 and leptin is based on a common structure, namely, their three-dimensional structure is similar and consists of four α-helices and two short β-threads [19]. The results of this study indicate an increase in gene expression and leptin secretion by adipocytes of the adipose tissue (AT) of different localizations in CAD. In the AT localized in the region of the heart, the expression and concentration of leptin increased to a greater extent compared with the SAT and was maximal in the EAT. It is believed that adipocytes are the only source of circulating leptin, and its activity at the local (tissue) level is provided by an interaction with leptin receptors (ObR), and that the SAT is the most active source of leptin [20]. Our data, on the contrary, indicated that the maximum expression of the leptin gene was observed in the EAT. It is likely that in CAD, adipocytes of EAT have an advantage for the synthesis of leptin.

The data obtained are consistent with the results of other studies that demonstrated an increase in the level of leptin mRNA in the EAT in patients with multivascular atherosclerotic lesions compared with patients with 1–2 CA lesions and those without CAD. Therefore, in patients with critical CAD in the EAT, significantly higher expression of leptin and IL-6 was observed than in SAT [14]. Zhang T. et al., described the relationship between the expression of leptin and coronary atherosclerosis, and showed that the level of leptin mRNA in the EAT in patients with stenosis was significantly higher than in patients without stenosis (p = 0.0431), which was in contrast to the SAT and serum leptin. Additionally, multivariate logistic regression analysis revealed that the level of leptin expression in the EAT was an independent risk factor for local CA stenosis (OR, 1.09; 95% CI, 1.01–1.18; p = 0.031) [21].

There is a scarcity of literature on the role of leptin in the PVAT. For example, Drosos I. et al. studied the expression of leptin in the PVAT surrounding the CA, in comparison with that surrounding the internal thoracic artery resistant to atherosclerosis [22]. The study found that *LEP* expression was increased in the PVAT surrounding the aortic root and the CA compared with the surrounding internal mammary artery, regardless of serum leptin levels. Additionally, the PVAT around the CA was characterized by more pronounced angiogenesis and inflammation, and a greater degree of fibrosis and hypoxia, which in turn enhances the transcription of the *LEP* gene and results in a significant increase in its mRNA levels [22]. According to our data, an increase in leptin in the PVAT took place only with an extremely severe degree of atherosclerotic vascular damage.

It should be noted that with an increase in the degree of atherosclerosis, considering the division of patients according to the severity of the CA lesion according to the SYNTAX Score, the highest gene expression and leptin concentration were observed in the severe CA lesions, compared to those with a moderate and extremely severe degree of CA lesion. The maximum expression was found in the EAT in individuals with severe CA lesions. The maximum expression of the *LEP* in the PVAT was observed much later and in patients with an extremely severe degree of CA atherosclerosis.

In addition to the pleiotropic effects that regulate metabolic, immune, and endocrine functions, leptin is undoubtedly involved in the pathogenesis of atherosclerosis, which is confirmed in both experimental and clinical studies [23]. Hyperleptinemia, associated with obesity, insulin resistance, and metabolic syndrome, is a predictor of the development of cardiovascular events, hemorrhagic stroke, and coronary restenosis after balloon angioplasty [24]. Additionally, leptin stimulates the production of pro-inflammatory cytokines by immune cells involved in atherogenesis, which in turn stimulate the synthesis of leptin *in vivo*, forming a positive feedback loop. The feedback loop provides the autocrine/paracrine effects of leptin, significantly increasing the risk of CAD and multivascular lesions of the CA. A study regarding the prevention of coronary diseases in Scotland (WOSCOPS) showed a moderate increase in a leptin associated risk of developing CAD [25].

The role of leptin in atherosclerosis was confirmed in an experiment on ob/ob mice with the absence of the atherosclerosis-resistant leptin gene, despite obesity and diabetes. The introduction of leptin led to atherosclerotic changes [26]. Available data on the overexpression of *LEP* in atherosclerotic plaques of the carotid arteries in patients with clinical symptoms suggest that locally produced leptin contributes to pro-atherogenic and remodeling processes leading to destabilization of atherosclerotic plaques [27]. Our results showed an increase in the prognostic significance of leptin in combination with adiponectin and the identification of the reciprocal effect between them.

In general, an increase in leptin in the AT localized in the epicardium and CAs is considered as an unfavorable factor associated with the progression of atherosclerosis. It is interesting to note that cardiomyocytes are capable of synthesizing leptin themselves and have leptin resistance, which is considered as a protective mechanism against the negative effects of this adipokine. Moreover, leptin can reduce myocardial contractility, which implies another possible relationship between the secretion of epicardial leptin and heart function [28].

### Study limitations

There were limitations to our study. First, it was a single-center retrospective study. Second, our sample size was small and there was no intermediate sensitivity analysis.

## 5. Conclusions

Thus, the results of the study indicate that an adipocytokine imbalance of the AT depends on its location. Therefore, adipocytes of the EAT in patients with CAD are characterized by a pronounced decrease in adiponectin mRNA against the background of increased leptin and IL-6. This feature of the EAT can have a negative effect on both adipocytes and cardiomyocytes, which possibly contributes to the aggravation of atherosclerotic processes and related complications.
